# Monitoring the impact of climate extremes and COVID-19 on statewise sentiment alterations in water pollution complaints

**DOI:** 10.1038/s41545-023-00244-y

**Published:** 2023-04-06

**Authors:** Anqi Liu, Jonghun Kam, Sae Yun Kwon, Wanyun Shao

**Affiliations:** 1 Division of Environmental Science and Engineering, Pohang University of Science and Technology (POSTECH), Pohang, 37673 the Republic of Korea; 2 Institute for Convergence Research and Education in Advanced Technology, Yonsei University, 85 Songdogwahak-Ro, Yeonsu-Gu, Incheon, 21983 the Republic of Korea; 3 Department of Geography, University of Alabama, Tuscaloosa, AL 35401 USA

**Keywords:** Water resources, Psychology and behaviour, Civil engineering

## Abstract

The COVID-19 pandemic and associated prevention policies can directly or indirectly alter the sentiment of individuals while registering water pollution complaints, but observational evidence remains limited. Here, we conducted a sentiment analysis on over 10,000 water pollution complaints from residents in Alabama, USA (2012–2021) to better understand how and to what extent COVID-19 has altered emotion (polarity score-based) and attitude (subjectivity) of water pollution complaints. We found that the 2017 state-wise drought significantly increased the percentage of negative water pollution complaints by +35%, with no significant alternation in attitude before the COVID-19 pandemic. Since COVID-19, the percentage of negative and subjective water pollution complaints significantly decreased and increased by −30 and +20%, respectively, and these sentiment alternations were maintained by 2021. This study provides a new direction for environmental governance and management, requiring a timely response to changes in the public’s emotions and attitudes during the next climate extremes and pandemics.

## Introduction

Since late 2019, the COVID-19 pandemic has resulted in the implementation of regulations and policies, limiting outdoor activities, such as social distancing, curfews, and quarantines. These prevention policies have affected the public perception of the risk of environmental incidents and natural disasters due to emotional and attitude changes (e.g., increased fear and anxiety)^1,2^. COVID-19 threatens our well-being and quality of life, including outdoor activity, sleep, time allocation, and mental health^3^. The risk of clinical depression has increased dramatically following the COVID-19 pandemic. According to a recent World Health Organization (WHO) report, anxiety and depression increased by 25% during the first year of the pandemic, which provoked more suicide and self-harm among young people^4^. These psychological effects of the COVID-19 pandemic can affect both the emotion and attitude of the public when they witness environmental and water pollution incidents, which requires a timely response from relevant authorities. However, understanding to which extent and how pandemics such as COVID-19 can alter sentiment in water pollution complaint reports is still lacking.

Besides the COVID-19 pandemic, climatic extremes such as floods and droughts can degrade water quality and cause severe water pollution incidents because of limited prediction skills^5^, resulting in the failure or misoperation of water-engineered systems^6^ and the absence of a timely water govenance policy^7^. These climatic extremes can also affect the mental health of the public directly and indirectly^8^. For example, loss of personal property and job instability following major floods and droughts cause psychological distress. Particularly, these droughts were a type of socioeconomic drought^9,10^. Sudden displacement and relocation force victims to build new social connections with neighbors and adapt to their new environment, which can also cause depression and anxiety^11^. During these climatic extremes, the public’s emotions and attitude toward degraded water quality and pollution can change, but observational evidence is limited.

Climatic extremes, such as floods and droughts, can threaten socioeconomic activities and the mental health of exposed societies^12–14^. When societies are exposed to unusual extreme events, the psychological impacts of these extremes can be amplified^15^. For example, the Apalachicola-Chattahoochee-Flint (ACF) River basin in the southeastern US region is a water-ample environment exposed to tornadoes, hurricanes, and thunderstorms, increasing the risk of floods and associated water pollution incidents. The ACF River basin has historically experienced persistent precipitation deficits and severe droughts^16,17^. These interstate water scarcities have threatened the southeastern US states’ continued growth and expansion. Finally, the recent interstate drought in 2017^18^ reignited water wars between the upstream state, Georgia, and the downstream states, Alabama and Florida^19^. Among these states, Alabama has the smallest budget for environmental management, with less than $11 per person in 2017 (the least budget among 46 US states)^20^. Interstate water scarcity and limited resources in Alabama can exacerbate the adverse impact of climatic extremes on water-related socioeconomic activities and mental health in vulnerable communities, requiring an efficient and effective environmental monitoring system for Alabamians’ well-being.

The Alabama Department of Environment Management (ADEM) has been operating an Internet web–based environmental pollution complaint platform, which can be used as a participatory surveillance system for environmental monitoring. Participatory surveillance systems have been widely adopted as cost-efficient and reliable monitoring systems in various fields, such as disease control^21^, marketing^22^, politics^23^, and urban planning^24^. Environmental and water pollution incident reports on social media and online platforms have become a common participatory surveillance system. For example, texts in air pollution complaints from the participatory surveillance systems provide new and reliable data to monitor participants’ sentiment^25^, which can monitor sentiment changes, indicating the need for authorities to reallocate financial and human resources accordingly. Participatory surveillance can also improve current water quality monitoring systems using human sensors without incurring significant costs^26^. However, studies of the utility of the public’s water pollution complaint reports is lacking.

Since June 2011, the ADEM has been posting public environmental complaints. Such long-term water complaints data from ADEM include reports during historical droughts and floods, and the COVID-19 pandemic, providing a unique research opportunity to the relative impact of COVID-19 on the public’s behavior patterns (e.g., emotion and attitude) when they report a water pollution complaint compared to historical climatic extremes before the COVID-19 pandemic. Therefore, this study uses text mining and sentimental analysis of more than 10,000 water pollution complaints to address the following questions:

1. How have historical climatic extremes such as drought and flood altered the public’s sentiment in water pollution reports before the COVID-19 pandemic?
2. What are the differences of the public’s sentiment in water pollution reports during the COVID-19 pandemic compared to historical climatic extremes?
3. To what extent have socioeconomic factors changed sentiment in the public’s water pollution reports during climatic extremes and the COVID-19 pandemic?

**Fig. 1:**
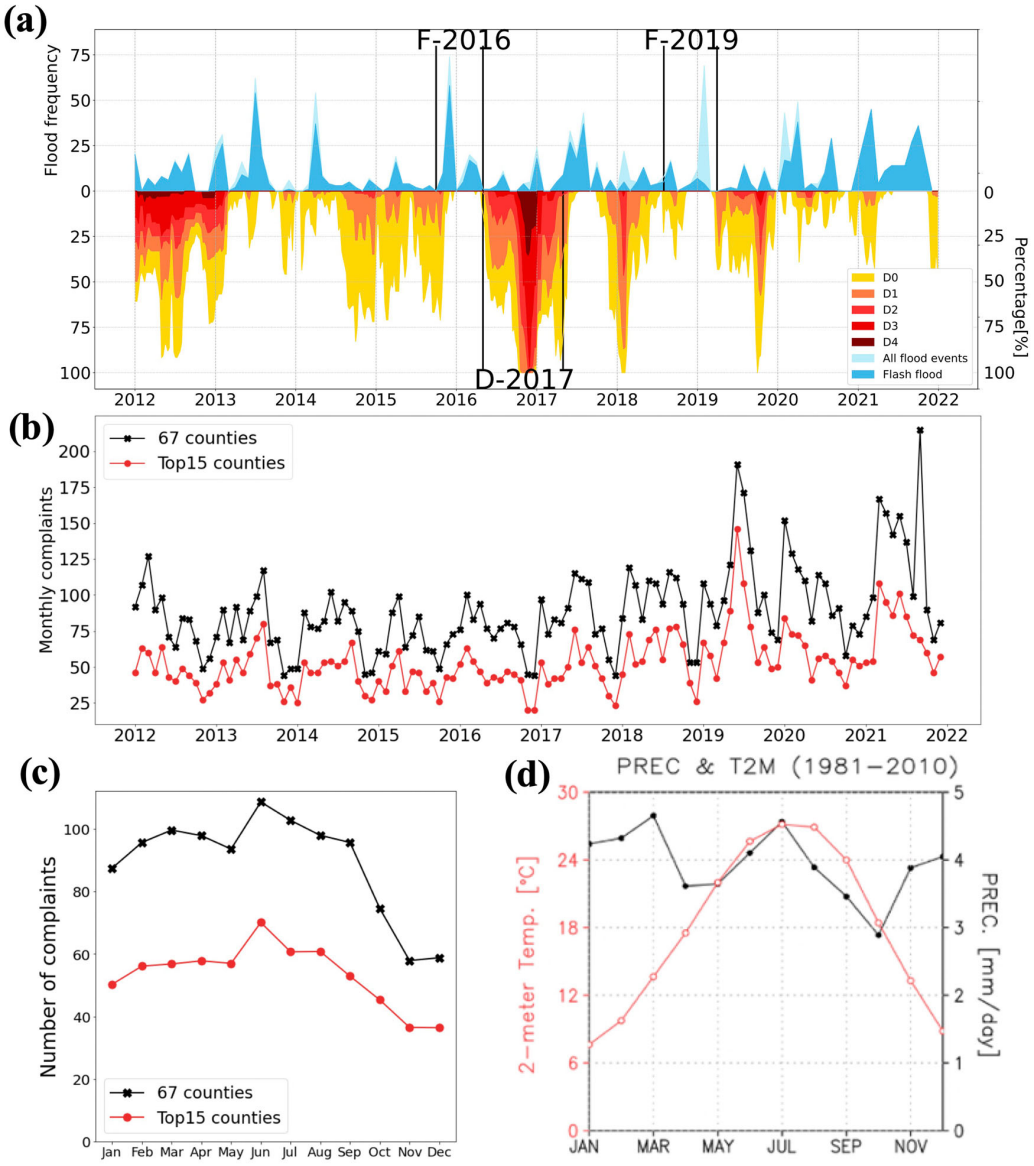
Occurrences of droughts and floods correlated with the trends and seasonality of water pollution complaints in Alabama. **a** D0, D1, D2, D3, and D4 depict the area fractions under abnormal dry, moderate drought, severe drought, extreme drought, and exceptional drought conditions, respectively. The blue color represents for the numbers of flood and flash flood events for 2012–2021. The black lines in depict the drought (D-2017) and two flood periods (F-2016, and F-2019). **b** Total number of monthly water pollution complaints from top 15 counties (red line with circles), and the entire state (black line with crosses). **c** Long-term averages for the numbers of complaints per month from the top 15 counties (red line with circles), and the entire state (red line with crosses) for 2012–2021. **d** Monthly average temperature (°C) (red line with empty circles) and precipitation (mm) (black line with solid circles).

To investigate the impact of climatic extremes before and after the COVID-19 pandemic, we first defined one drought period and two flood periods based on the extent and magnitude of the detected droughts by the U.S. Drought Monitor and the frequency of the detected floods by the National Oceanic and Atmospheric Administration (see *Methods*). The drought period was 2016–2017 (D-2017 in Fig. 1a), with two flood years in 2016 and 2019 (F-2016, and F-2019 in Fig. 1a). During D-2017 (see Supplementary Fig. 1), 80% of Alabama experienced abnormally dry to extreme drought conditions. While F-2019 showed a high frequency of riverine floods (i.e., river inundation-driven), F-2016 showed high frequencies of flash floods (i.e., precipitation-driven). A riverine flood occurs only along the river streamline, whereas a flash flood occurs at any land surface within six hours of heavy rainfall^27^. We also defined 2020 and 2021 as the COVID-19 pandemic period.

Secondly, we performed a significance test of the impact of climatic extremes and COVID-19 on sentiment alteration in emotion and attitude in the public water pollution complaints by comparing changes in the percentage of negative/positive complaints (emotion) and subjective/objective complaints (attitude) compared to the corresponding percentage of randomly sampled complaints (with replacement; bootstrapping) over the entire study period (Refer to the “Methods” section). A percentage ratio of unity (1.0) means no change. If the 95th percentile range is above/below unity for a certain period, it indicates that a statistically significant change has occurred (Refer to the “Methods” section). To further investigate the impact of socioeconomic development, we separated complaints from residents of the top 15 counties in terms of GDP and the other 52 counties.

## Results

### Variations and trends in water pollution complaints (2012–2021)

Annual total water pollution complaints showed no significant changes over 2012–2018 (Fig. 1b). Complaints led the peak in 2019 was led by complaints from the counties of Cullman and Walker, while the second peak was led by Perry, which had the 10th-, 13th- and 16th-most water pollution incidents, respectively. Together, these two counties had 72 and 49 complaints in June and July 2019, respectively (Supplementary Fig. 2), because of local polluted water discharges that killed fish in the Black Warrior River^28^, and Perry had 104 in September 2021. Water pollution complaints reports increased dramatically in June 2020, reaching 196 reports for the month before decreasing slightly in the rest of 2020. In 2021, the number of complaints increased again in the summer and reached 215 in September. From 2012 to 2021, the top 15 counties in terms of GDP (i.e., 70% of Alabama’s GDP in 2019) contributed approximately half of the complaints. The result indicates that the public’s complaints are a reliable source for monitoring water pollution. The 262 water pollution complaints in 2019 and 2021 were excluded from our assessment (Supplementary Fig. 3) to investigate the impact of the COVID-19 pandemic and climatic extremes, rather than the impact on water pollution incidents.

The long-term monthly average of water pollution complaints showed a seasonal pattern throughout the year. Water pollution complaints peaked at ~100 from January to August and gradually decreased from September to ~60 complaints in December (Fig. 1(c)). Complaints increased during the wet season (January–August), and the gradual decrease from September to December matched the decrease in the monthly temperature (Fig. 1(d)). These indicate a combined effect of precipitation and temperature on the frequency of the public’s observation of/exposure to water quality degradation. In addition, residents in the counties with higher population density and GDP have reported more water pollution complaints (Supplementary Fig. 4 and Supplementary Table 1).

### Influences of climatic extremes before COVID-19

**Fig. 2:**
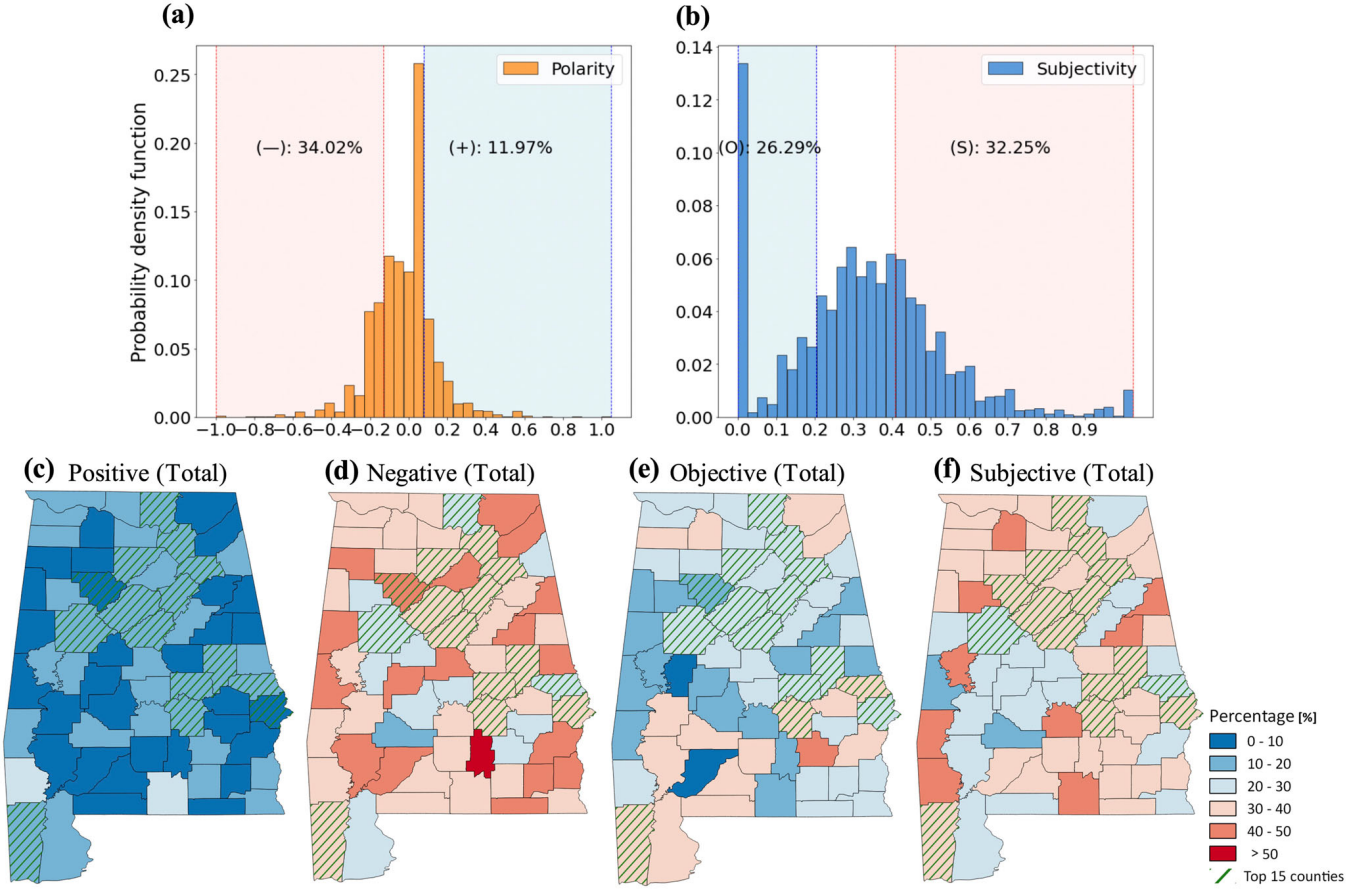
Spatial patterns of the sentiment of water pollution complaints over 2012–2021. Probability mass functions of **a** polarity and **b** subjectivity scores. The percentages of positive (+), negative (−), objective (O), and subjective (S) complaints are shown in the text. County-level maps of the percentages of positive (**c**), negative (**d**), objective (**e**), and subjective (**f**) complaints. Green-colored hatch counties depict the top 15 counties in Alabama in terms of GDP in 2019.

Over 2012–2021, 12 and 34% of the public’s water pollution complaint reports showed positive (polarity score ≥ 0.1) and negative emotion (polarity score ≤ −0.1), respectively, at the state level (Fig. 2a). Twenty-six and 32% of the water pollution complaint reports showed subjective (subjective score ≥ 0.4) and objective (subjective score ≤ 0.2) attitude, respectively (Fig. 2b). Overall, residents in the top 15 counties posted more objective and less negative complaints than residents in the other 52 counties (green-colored hatched counties in Fig. 2c–f).

**Fig. 3:**
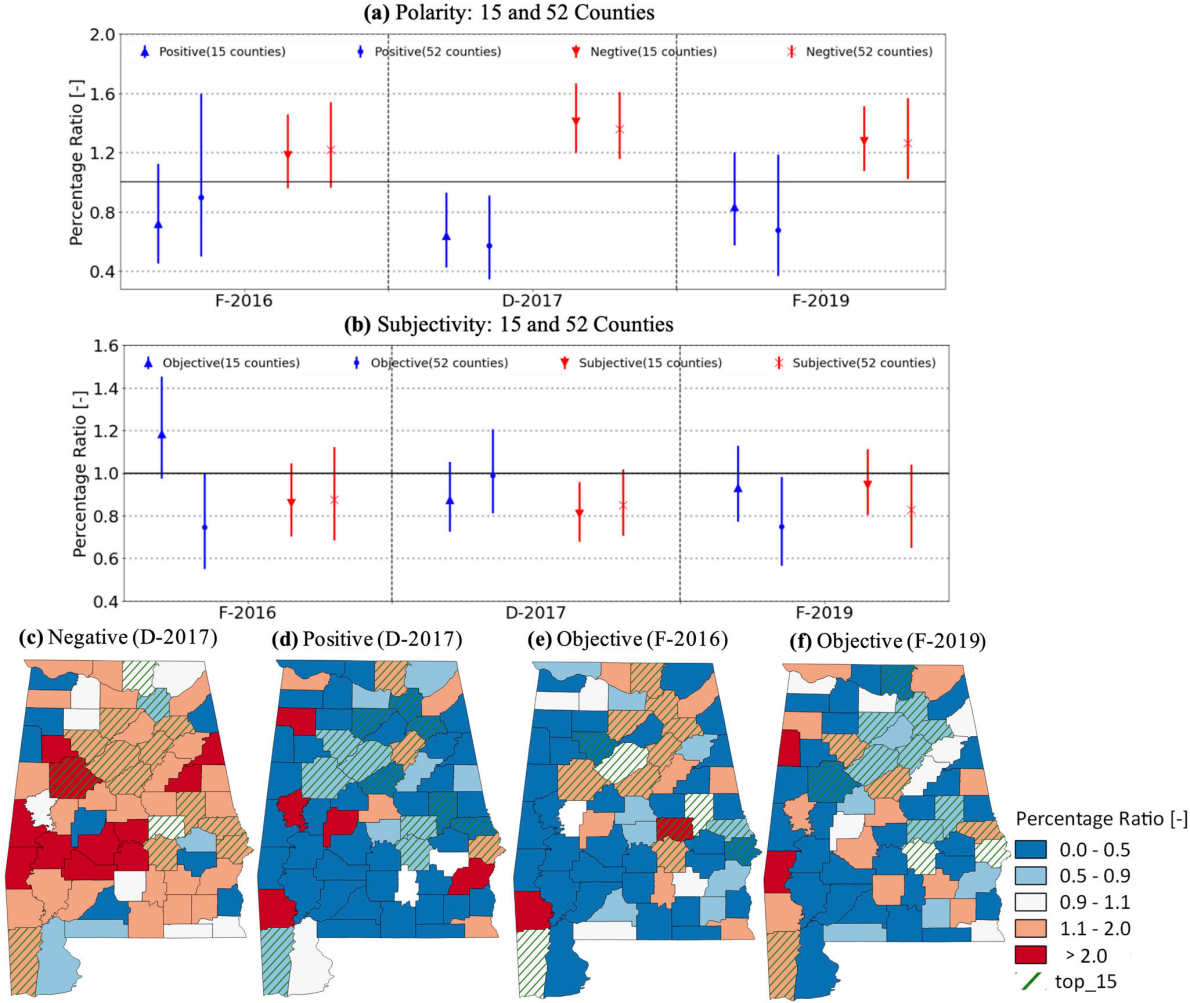
Altered sentiment of water pollution complaints during historical climatic extremes. Triangles and downward triangles (circles and cross markers) in (**a**) depict the percentage ratios of positive and negative complaints, respectively, based on polarity scores to the state-level percentages for the top 15 (other 52) counties (other 52 counties). Triangles and downward triangles (circles and cross markers) in (**b**) depict the percentage ratios of objective and subjective complaints, respectively, based on subjectivity scores to the state-level percentages for the top 15 (other 52) counties. Error bars depict the 95th percentile ranges of the percentage ratios from 10,000 bootstrapped samples. County-level maps in (**c**) and (**d**) depict the percentage ratios of negative and positive complaints, respectively, during D-2017. County-level maps in (**e**) and (**f**) depict the percentage ratios of objective complaints during F-2016 and F-2019, respectively.

During F-2016, water pollution complaints show no significant changes in emotion and attitude in top 10 and other 52 counties (Fig. 3a, b, e). During F-2016, a majority of floods were flash/pluvial floods (Fig. 1a), indicating a weak impact of flash flood on alteration in emotion and attitude of water pollution complaints.

During D-2017, more water pollution complaints show negative emotion at the state level by increasing their percentages by +35% (95th percentile range (hereafter, CI95): +15 to +60%) of negative complaints over the entire study period. Other 52 counties showed a higher percentage of negative complaints than the top 15 counties (Fig. 3(c)). Furthermore, more water pollution complaints show a neutral attitude by decreasing the percentage of subjective and objective complaints by −10%. D-2017 was a flash drought typically characterized by a rapid onset and intensification over a broad area^29^. In June 2017, the drought was centered in northeastern Alabama, and severe drought conditions spread to over 80% of the state within four weeks (Supplementary Fig. 1). This indicates that the sudden emergence of a flash drought can significantly alter both the emotion and attitudes of water pollution complaints.

Residents in the top 15 and other 52 counties reported more negative and less subjective water pollution complaints during F-2019, changing their percentage by +25% (CI95: +3–50%) and −25% (CI95: −1 to 40%) respectively, than those during the entire study period. F-2019 was a type of riverine/fluvial flood, indicating a significant impact on the alteration of emotion and attitude in water pollution complaints.

Despite no significant alternation of attitude during D-2017, the top 15 counties had fewer objective complaints during the two flood periods than during the entire study period (Fig. 3(e) and (f)). The results indicate that the type and severity of climatic extremes alter emotions and attitudes toward water pollution complaints differently, requiring a further investigation of the dynamics and processes for sentiment alternation during droughts and floods at the individual level via interview/survey with a careful questionnaire design.

### Influences of COVID-19

**Fig. 4:**
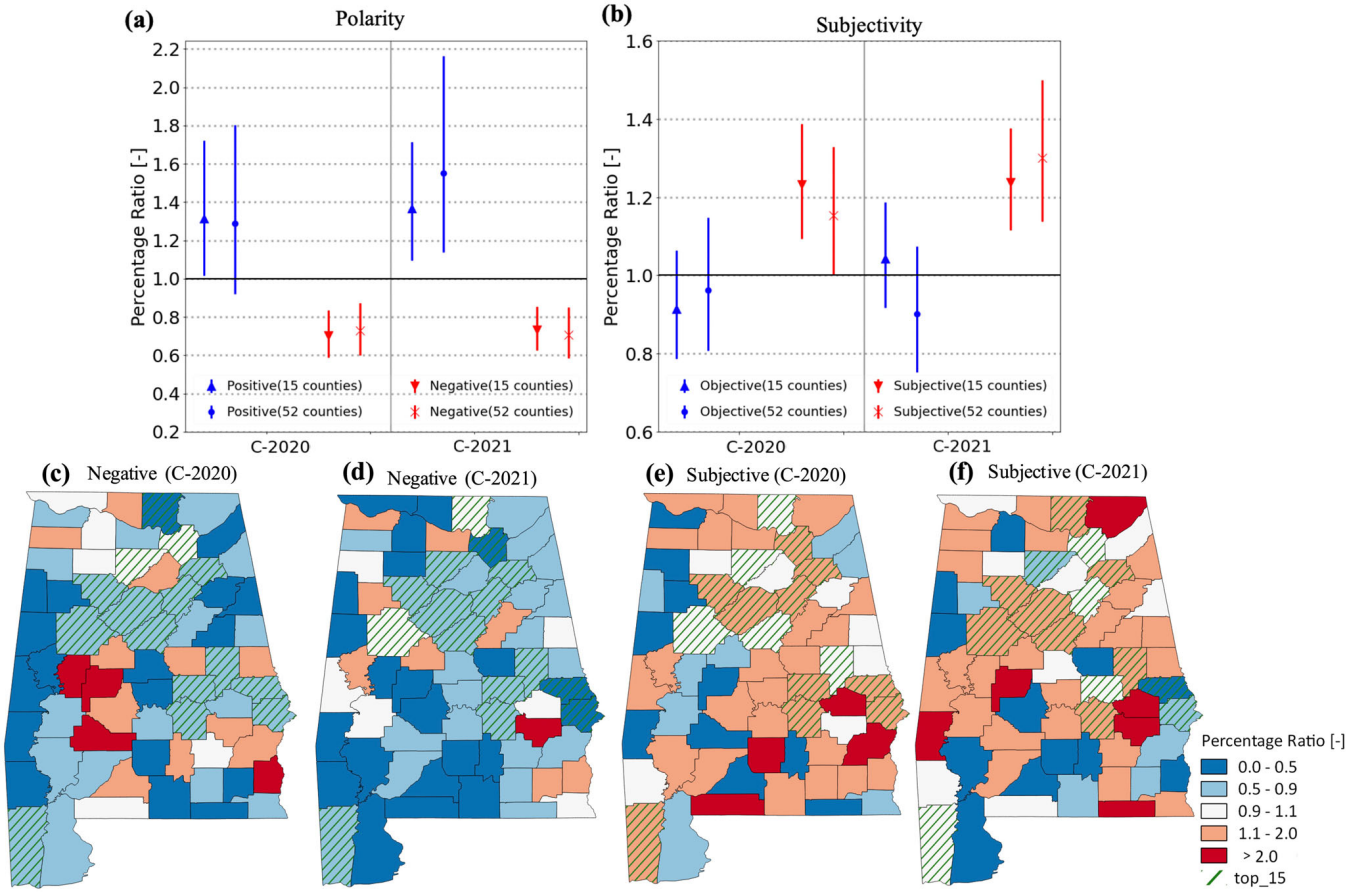
Altered sentiment of water pollution complaints during the COVID-19 pandemic. Triangles and downward triangles (circles and cross markers) in (**a**) depict the percentage ratios of positive and negative complaints, respectively, based on polarity scores to the state-level percentages for the top 15 counties (other 52 counties) during 2020-21. Triangle and downward triangles (circles and cross markers) in (**b**) depict the percentage ratios of objective and subjective complaints, respectively, based on subjectivity scores to the state-level percentages for the top 15 (other 52) counties during 2020-21. Error bars depict the 95th percentile ranges of the percentage ratios complaints from 10,000 bootstrapped samples. County-level maps in (**c**, **d**) and (**e**, **f**) depict of ratios of the percentage ratios of negative (subjective) complaints during 2020-21.

In 2020, the percentage of negative complaints decreased remarkably by state (approximately −30% (CI95: −40% to −20%); Fig. 4a). The percentage of subjective complaints increased significantly at the state level, with a big difference between the top 10 and other 52 counties (+20% (CI95: +10% to +38%) and +15% (CI95: +1% to +34%), respectively). The results indicate a significant psychological impact of COVID-19, particularly the prevention policies, on the public’s reporting behavior patterns about water pollution. The COVID-19 lockdown significantly constrained both industrial and social activities, reducing water pollution^30^. Furthermore, COVID-19 prevention policies restricted outdoor activities by the public, which reduced their exposure to visible water pollution issues. COVID-19 also took a mental toll on people, and the amount of attention/concern of humans has been analogized as “a limited pool”^21^. In other words, much of the public’s concern shifted to public health since the COVID-19 pandemic.

In 2021, the emotion and attitude alterations continued, indicating the long-term impact of COVID on emotion and attitude alternation in water pollution complaints. Interestingly, few of the other 52 counties show significantly increased negative and subjective complaints, indicating a possible impact of the socioeconomic structures. The COVID influence on economic activities and education level may have altered sentiment (emotion and attitude) differently, requiring a better understanding of how and to what extent the socioeconomic structure of exposed communities can alter emotion and attitude in risk communication, particularly during the COVID-19 pandemic.

## Discussion

We found that since the COVID-19 pandemic, socioeconomic development has played a significant role differently in the sentiment alteration of water pollution complaints. The COVID pandemic altered sentiment across the counties in 2020 and 2021. Since COVID-19, both the top 15 and the other 52 counties have seen an increase in positive and subjective complaints while decreasing in negative complaints. This analysis of sentiment alternations offers assistance to the government to better monitor public emotional changes on water pollution, thereby reducing further pollution events, preventing extensive negative emotions among the public, and even causing unrest.

We found that socioeconomic development played different roles in the sentiment (emotion and attitude) of the water pollution complaints, depending on the types of climatic extremes such as droughts and floods. Before COVID-19, D-2017 and F-2019 altered emotions and attitudes in water pollution complaints, whereas changes in emotions and attitudes were not significant during F-2016. This finding indicates that a better understanding of the characteristics of droughts and floods can help authorities better manage residents’ complaints. Furthermore, emotion alternation is more dependent on the socioeconomic development of affected communities, with a minor impact on attitude alternation, indicating observational evidence of the impact of different demographic characteristics of exposed communities on risk communication.

This study demonstrated observational evidence that sentiment alternation of water pollution complaints was diverse during the climatic extremes and the COVID-19 pandemic. The remaining key factor for the changes in sentiment is the severity of the water pollution incident itself. While the data used in this study have limited information about the severity, we conducted an additional sentiment analysis on a relatively small volume of water pollution complaints in 2019 and 2021 (Supplementary Fig. 2). The environmental incidents significantly increased the percentage of positive water pollution complaints (Supplementary Fig. 5), indicating a comparable impact of environmental incidents on emotions and attitudes of water pollution complaints.

Generally, the “shelf life” of emotion can be shorter than that of attitude. Emotions can be changed dramatically, which is related to not only what the public was going through at the time, but also how they felt. On the contrary, income and educational level may influence attitudes measured by “subjectivity” scores, which are more related to how to observe and report an environmental incident, using long-term education experience^31^. More objective complaint reports can improve the reliability and practicability of participatory surveillance in water pollution monitoring. Therefore, the findings of this study highlight the importance of public outreach and education programs, which can help in water resource monitoring and management through participatory surveillance. Furthermore, we found diversity in the potential mechanisms for the sentimental alternation in risk communication during climate extremes and COVID-19, which requires interdisciplinary collaboration between natural and social scientists leveraging big data.

The lack of available water resources adversely affects the well-being of our lives, including sanitation, infectious disease, and mental health of the public. People who face water scarcity and insecurity will experience higher anxiety, depression, and stress, especially in rural areas. These passive emotions can cause frustration and interpersonal conflicts^32^. The spread of negative emotions may reduce their initiative to improve the local environment and even cause demonstrations or personal vindictive acts, resulting in property losses and environmental damage^33^, which is an interaction of available water resources (water quantity and quality) and mental health. This study examines the impact of climatic extremes and pandemics on sentiment alternation in water pollution complaint reports.

Finally, this study emphasizes the utility and practicability of digital technologies in water quality monitoring, such as text mining and sentiment analysis tools, in guiding a direction toward smart environmental governance and management. This study suggests that the monitoring data from participatory surveillance has a potential value in opening new questions for surveys and interviews, which can further explore the detailed mechanisms of observed sentiment alteration in water pollution complaints. Traditional and digital technologies combined can advance our understanding of the interconnections of sentiment alternation at multiple scales (e.g., individual to state level) and across the different pollution mediums (e.g., water, air, and land). This study emphasizes the importance of the citizen-participant (bottom-up) strategy of current operational monitoring systems in Alabama, a vulnerable state to water scarcity, which can bring both short- and long-term successes to integrative watershed management for water security and satisfaction with complaint resolution, which can be used to resolve water scarcity and security in other states and nations.

## Methods

### Data

The water pollution complaints are publicly available data, and they were retrieved from the website of the ADEM; https://app.adem.alabama.gov/complaints/default.aspx. Data on extreme events were acquired from different institutions: the drought severity data were taken from the US Drought Monitor (https://droughtmonitor.unl.edu/Data.aspx), the flood frequency data were taken from the National Oceanic and Atmospheric Administration (NOAA; https://coast.noaa.gov/digitalcoast/data/home.html), and new confirmed cases of COVID-19 were taken from the COVID Tracking Project (https://covidtracking.com/data/download). The data were used to identify extreme events such as droughts, floods, and COVID-19 and the social response.

### Collection of complaint data

10,690 water pollution complaint reports from 67 counties in Alabama for the period of 2012–2021 were collected from the Alabama Department of Environmental Management website (https://app.adem.alabama.gov/complaints/default.aspx) through BeautifulSoup^34^. Water pollution complaints from 2012 to 2020 were retrieved on July 20, 2021. Water pollution complaints in 2021 were retrieved on July 4, 2022. Each complaint contained the county name, complaint ID, complaint time, complaint method, and detailed content. Supplementary Table 2 presents an example of the complaint data. These complaints were reported through various methods: phone calls (45.46%), web (39.75%), email (13.27%), mail (0.96%), in-person (0.54%), and fax (0.02%).

### Identification of periods of climatic extremes and pandemic

To investigate the public’s response to water pollution and the impacts of certain events, periods corresponding to droughts, floods, and the COVID-19 pandemic were defined. Drought periods were defined based on the drought index provided by the US Drought Monitor: D0, D1, D2, D3, and D4 correspond to abnormally dry, moderate drought, severe drought, extreme drought, and exceptional drought, respectively (Fig. 1a). The total areal percentages of Alabama under D0–D4 conditions were used to determine the level of drought severity (e.g., 100 means the entire state is under drought conditions). One apparent drought period with high areal percentages under the D4 condition were detected: May 2016 to April 2017 (D-2017).

The flood frequency in each month from 2012 to 2021 is shown in Fig. 1a. Over the study period, three periods with high flood frequencies were defined: October 2015–April 2016 (F-2016), and August 2018–July 2019 (F-2019). The maximum monthly flood frequency was 74 in December 2015 during F-2016. The second-highest flood frequencies were 69 in February 2019 during F-2019.

The COVID-19 pandemic was defined as taking place from January 2020 to December 2021, with year 2020 called C-2020 and year 2021 named C-2021 in this study. Data from the COVID Tracking Project showed that the new confirmed cases in Alabama increased continuously from March 2020, and experienced two peaks until December 2021.

### Text mining and sentiment analysis

The most frequent words in textual data are commonly extracted to mine the opinion hidden in the text. To eliminate noise, meaningless context and invalid characters were excluded, such as prepositions, verbs, and names^35^.

In this study, TextBlob was used to compute the polarity and subjectivity scores of the water pollution complaints reports^36^. The polarity scores can determine whether the text implies a positive, negative, or neutral opinion and ranges from −1 (extremely negative) to 1 (extremely positive). The subjectivity scores indicates whether a sentence contains a subjective opinion or objective fact and ranges from zero (objective fact) to one (highly subjective opinion)^37^.

To investigate the relative changes in complaints during climatic extremes and the COVID-19 pandemic, we first randomly sampled with replacement (i.e., bootstrapping) complaints during the periods of droughts, floods, and the COVID-19 pandemic 10,000 times. Then, we calculated the percentages of complaints with positive and negative scores to the total complaints of the 10,000 randomly sampled sets. Finally, we found the corresponding percentages to the 2.5th, 50th, and 97.5th percentiles among the 10,000 samples. For the reference, we randomly sampled with replacement the complaints during the study period and found the corresponding percentages to the 2.5th, 50th, and 97.5th percentiles among the 10,000 bootstrapping samples.

## Supplementary information

Supplementary Material

## Data Availability

Water pollution complaint report data and python codes for data collection and sentimental analysis used in this study are available at 10.7910/DVN/7N7GTE. Drought areal extent data and Flood frequency data for the US state of Alabama is available at the US drought monitor (https://droughtmonitor.unl.edu/) and the National Centers for Environmental Information (NCEI) - NOAA (https://www.ncei.noaa.gov/pub/data/swdi/stormevents/csvfiles/).
