# Supplementary material for: Monitoring the impact of climate extremes and COVID-19 on statewise sentiment alterations in water pollution complaints
Source: NPJ Clean Water. 2023 Apr 6;6(1):29. doi: 10.1038/s41545-023-00244-y (PMC10079150; doi:10.1038/s41545-023-00244-y)
Supplement: Supplementary file 1 — Supplementary Material [file 41545_2023_244_MOESM1_ESM.docx]

Supplementary Materials for

**Monitoring the impact of climate extremes and COVID-19 on statewise sentiment alterations in water pollution complaints**

Anqi Liu^1^, Jonghun Kam^1^*, Sae Yun Kwon^1^, Wanyun Shao^2^

^1^Division of Environmental Science and Engineering, Pohang University of Science and Technology, Pohang, 37673 South Korea

^2^Department of Geography, University of Alabama, Tuscaloosa, AL 35401, United States

**Re-submitted to *npj Clean Water***

**March 3, 2023**

Supplementary Materials include:

Supplementary Table 1 & 2

Supplementary Figure 1-5

**Supplementary Table 1. Data sources**

| **Parameter** | **Source** | **Time span** | **Webpage link** |
| --- | --- | --- | --- |
| Drought | US Drought Monitor | 2012-2020 | https://droughtmonitor.unl.edu/CurrentMap.aspx |
| Flood | NOAA | 2012-2020 | <https://coast.noaa.gov/digitalcoast/data/home.html> |
| COVID | The COVID Tracking group | 2020 | <https://covidtracking.com/data/state/alabama> |
| WWTPs | ADEM (Alabama department of environmental management) | 2021 | <https://gis.adem.alabama.gov/mun/index.html> |
| GDP | Bureau of Economic Analysis (BEA, US Department of commerce) | 2016-2019 | <https://www.bea.gov/> |
| Education | USDA (US Department of Agriculture) | 2019 | <https://www.ers.usda.gov/data-products/county-level-data-sets/download-data/> |
| Poverty | USDA (US Department of Agriculture) | 2019 | <https://www.ers.usda.gov/data-products/county-level-data-sets/download-data/> |
| Income | USDA (US Department of Agriculture) | 2019 | <https://www.ers.usda.gov/data-products/county-level-data-sets/download-data/> |
| Population | USDA (US Department of Agriculture) | 2019 | <https://www.ers.usda.gov/data-products/county-level-data-sets/download-data/> |

**Supplementary Table 2. Example of public complaints data**

| **County** | **Complaint ID** | **Datetime** | **Complaint Method** | | **Description** |
| --- | --- | --- | --- | --- | --- |
| JEFFERSON | 3F-007GE1L07 | 2011/6/1 12:00 | Phone | Neighbor dumping oil on ground. A creek is nearby. | |
| LIMESTONE | 7N-003GX5W41 | 2011/6/1 12:00 | Web | A soil/gravel borrow pit has been abandoned on Old School House Road and has not been restored to its original state. | |
| MOBILE | 4X-005GA6Y13 | 2011/7/7 12:00 | Email | An unpermitted site close to an acre of disturbance is abutting a tidal emergent marsh system of Dog River. A rain event will in all likelihood cause a water quality concern by the washing of recently disturbed soils in the marsh and water column. No BMPs were present. Construction equipment was active. | |


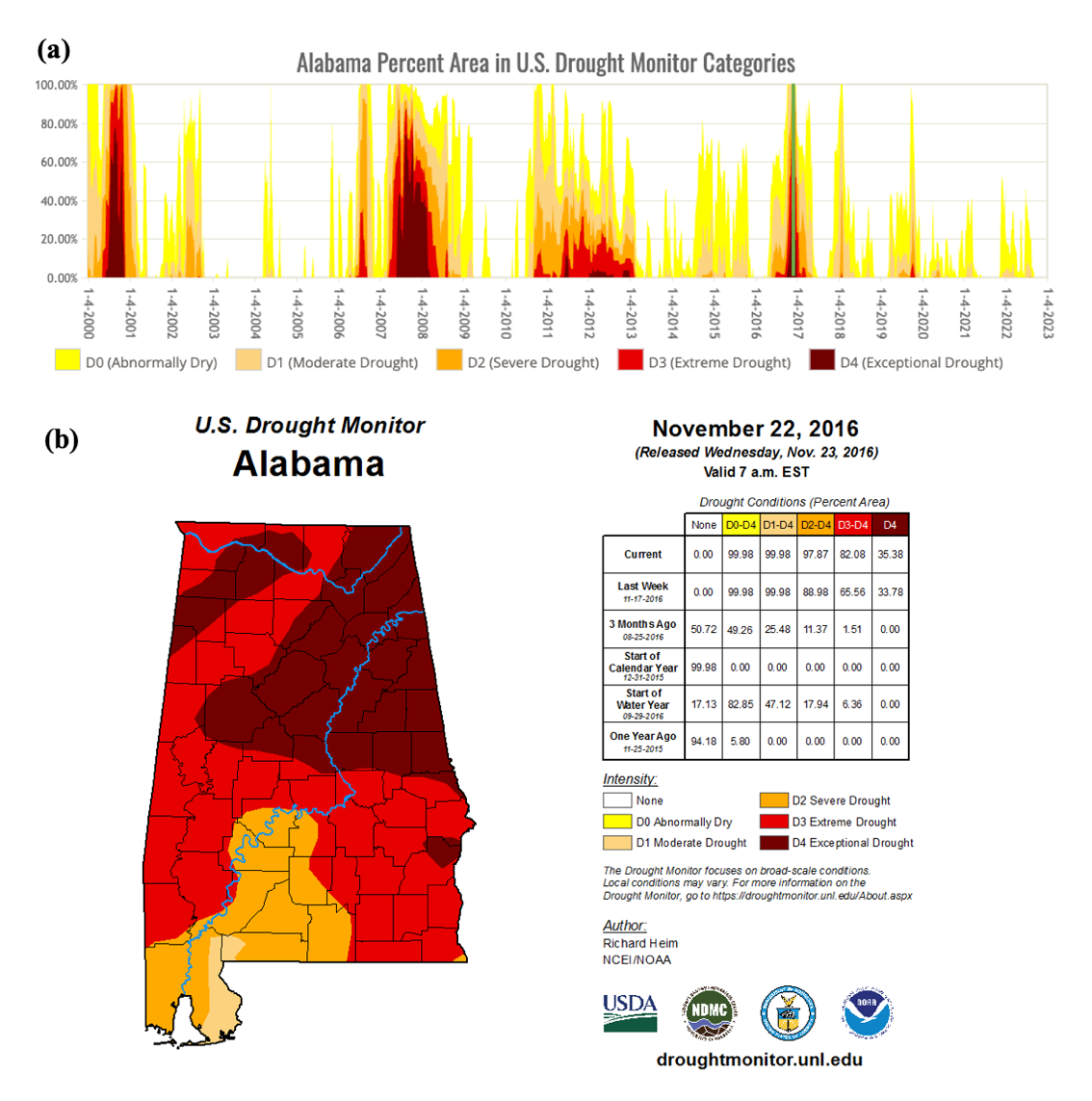


**Supplementary Figure 1.** Spatial extent of droughts during the 2017 drought period in **Fig. 1**. This figure is created by modifying the figures that are retrieved on March 16, 2022 from the US Drought Monitor website (<https://droughtmonitor.unl.edu/>).


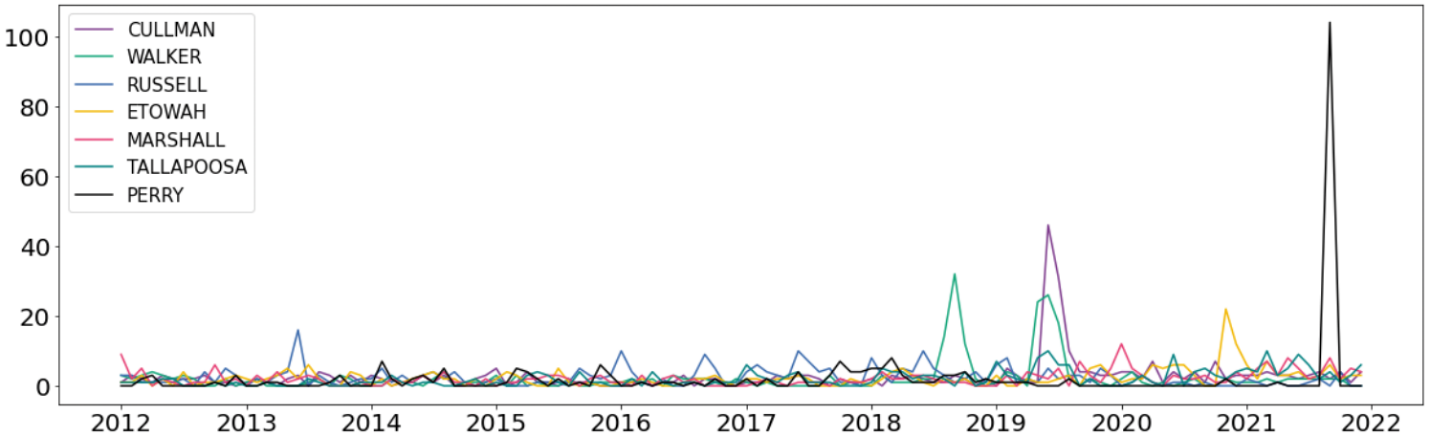


**Supplementary Figure 2. Monthly complaints for the top 10 to 16 counties.** Purple, light blue, blue, light green, green, pink and black color lines with circles depict top 10 (Cullman), top 11 (Russell), 12 (Etowah), 13 (Walker), 14 (Marshall), 15 (Tallapoosa), 16 (Perry) counties with the most complaints and 67 countries, respectively.


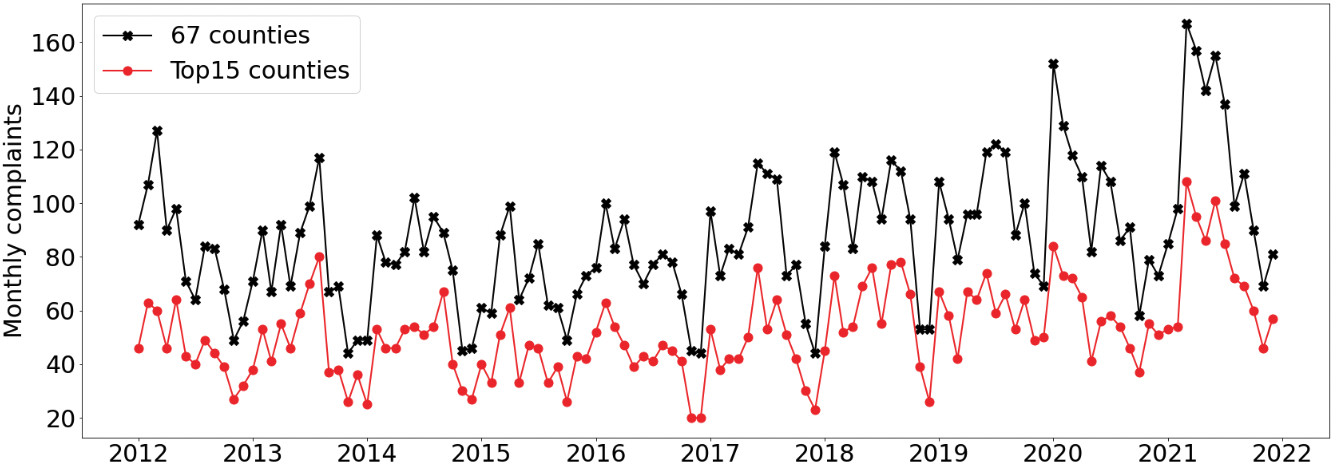


**Supplementary Figure 3. Numbers of complaints per month after removing outliers.** Blue and red color lines with circles depict top 15 counties with the most complaints and 67 countries, respectively.


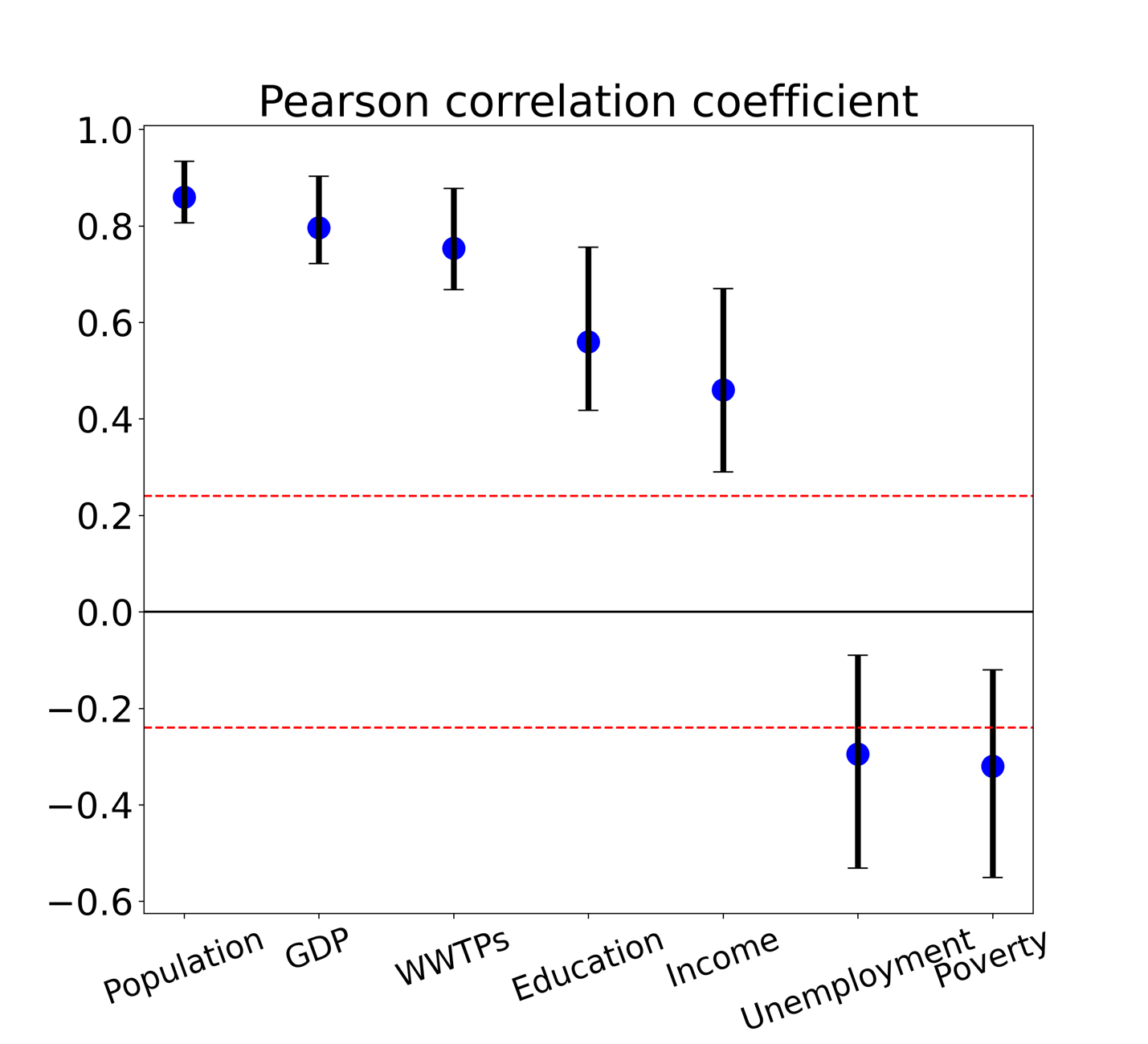


**Supplementary Figure 4. Pearson correlation coefficients between the number of complaints and socioeconomic factors.** Error bars depict the 95th percentile range of Pearson correlation coefficients.


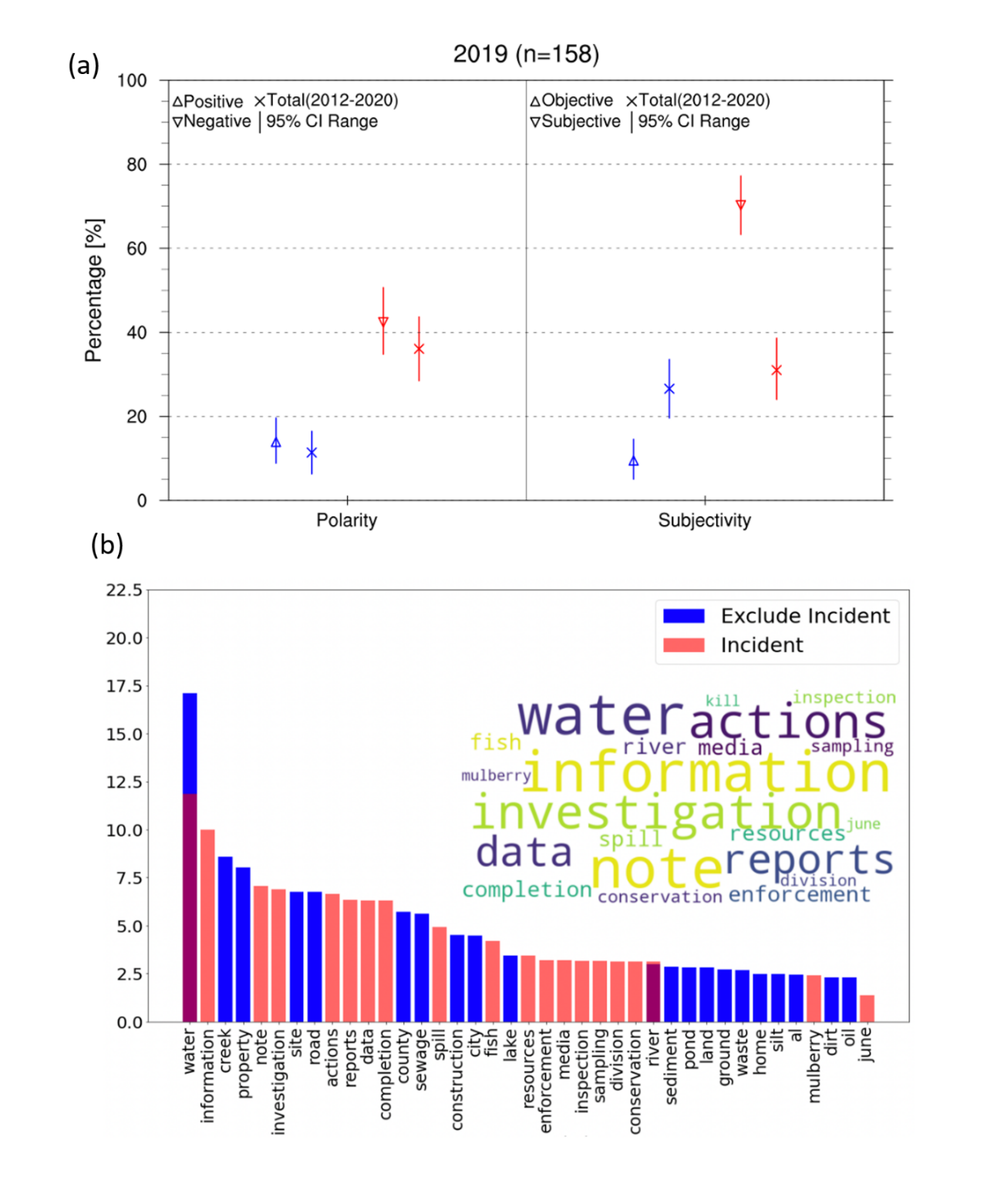


**Supplementary Figure 5. Impact of the 2019 environmental incidents on the sentiment of water pollution complaints.** (a) Percentages of positive (triangle), negative (upside down triangles), objective (triangle), and subjective (upside down triangles) water pollution complaints during the 2019 environmental incident. (b) The top 20 words that were most frequently used during the 2019 environment incident (red bars) and the entire study period (blue bars). The y-axis depicts the percentage of the frequency of keywords to the total frequency of the top-20 words (i.e., 100 × (frequency of a keyword/ total frequency of all 20 words). The 20 most frequently words are shown as a word cloud.
